# Imaging Findings of the Distal Radio-Ulnar Joint in Trauma

**DOI:** 10.5334/jbr-btr.846

**Published:** 2015-09-15

**Authors:** M. Mespreuve, F. Vanhoenacker, K. Verstraete

**Affiliations:** 1 St.-Maarten General Hospital, Leopoldstraat 2, 2800 Mechelen, Belgium; 2 University Hospital Ghent, Belgium; 3 University Hospital Antwerp, Belgium

**Keywords:** Wrist, injuries

## Abstract

Traumatic lesions of the distal radio-ulnar joint (DRUJ) occur frequently in conjunction with fractures of the distal radius. They are a common cause of pain and limited range of motion after distal radial fractures. Due to the complex anatomy they are however often ignored or underappreciated. Distal radial fractures and luxations of the DRUJ often disturb the normal curvature of the radial notch and cause damage to the cartilage of this joint. The growth of the radius may be disrupted, resulting in a positive ulnar variance, and possibly give rise to complications such as ulnar abutment and motion restriction.

Ulnar styloid fractures – sometimes barely visible on plain film – may give rise to symptomatic bony pseudarthrosis, dislocation and laceration of the tendon of the m. extensor carpi ulnaris and a rare posttraumatic deformity of the ulnar epiphysis. Also the possibility of lesions at the adjacent triangular fibrocartilage complex and the joint capsule should be kept in mind.

This paper presents a pictorial review of the complex functional anatomy and pathologic conditions of this joint and emphasises why the DRUJ should be evaluated independently and thoroughly. The merit of each imaging modality is mentioned.

A correction article relating to Fig. 2 and Fig. 27 can be found here: http://dx.doi.org/10.5334/jbr-btr.966

## Publisher's Note

A correction article relating to Fig. 2 and Fig. 27 can be found here: http://dx.doi.org/10.5334/jbr-btr.966

## Introduction

Traumatic lesions of the distal radio-ulnar joint (DRUJ) occur frequently in conjunction with fractures of the distal radius and are a common cause of pain and limited range of motion after distal radial fractures. However they are often ignored or underappreciated. Therefore this joint should be evaluated independently and thoroughly.

The aim of this pictorial review is to present an overview of the spectrum of traumatic disease of the DRUJ and to highlight the role of imaging.

## Anatomy and biomechanics

**Figure 1:**
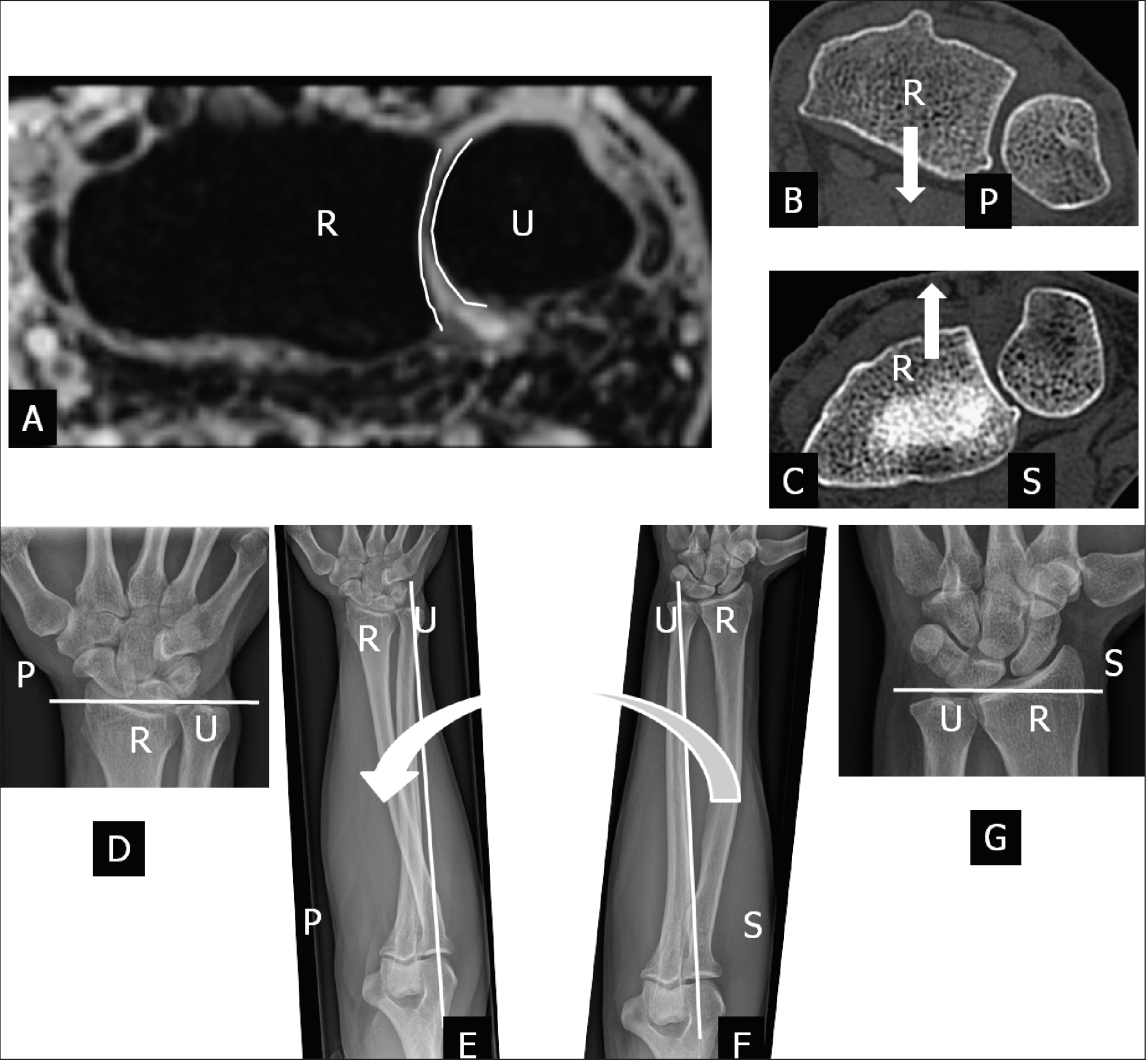
The normal curvature and motion of radius (R) and ulna (U) at the cylindrical cartilaginous DRUJ. Axial 3D-GRE image (A), axial CT-scan (B–C) and plain radiography (D–G). A. The radial sigmoid notch is a shallow concavity found along the ulnar border of the radius. This sigmoid notch (left white curved line) has a greater radius of curvature than the more convex ulnar head (right white curved line). B. During pronation (P) the radius translates palmarly (white arrow) relative to the distal ulna. C. During supination (S) the radius translates dorsally (white arrow) relative to the distal ulna. D–E. During pronation (large curved arrow) the radius axially shortens relative to the distal ulna. The axis of forearm motion (white vertical line) is running through the proximal head of the radius and the distal head of the ulna. The radius is rotating around the ulna, which remains fixed. F–G. The reverse motion occurs during supination (S) as the radius lengthens in relation to the ulna.

The distal radio-ulnar joint (DRUJ) is composed of the radius and ulna, the triangular fibrocartilage complex (TFCC) and the joint capsule. The radial sigmoid notch is a shallow concavity found along the ulnar border of the radius. The shape of the sigmoid notch varies from C-shaped to flat and S – shaped. This sigmoid notch has a greater radius of curvature than the more convex ulnar head (Fig. 1A) [1].

The pronation-supination of about 150–160 degrees at this joint is a combination of translation (Fig. 1B–C) between the radial sigmoid notch and ulnar head and rotation (Fig. 1D–G) between the radius and ulna [2, 3]. The longitudinal axis of forearm rotation is passing through the center of the head of the radius proximally and the foveal sulcus at the base of the ulnar styloid distally [44]. During this motion the radius, the carpus, and the TFCC rotate over the fixed ulnar head (the ulna is unable to rotate as the humeroulnar hinge-joint at the elbow allows only flexion and extension). During pronation the radius axially shortens (Fig. 1D–E) and translates palmarly (Fig. 1B) relative to the distal ulna. The reverse motion occurs during supination as the radius lengthens (Fig. 1F–G) in relation to the ulna and translates dorsally (Fig. 1C) [3, 4, 5].

## Clinical manifestation of DRUJ dysfunction

DRUJ injuries can give rise to long lasting complaints. Restricted and/or painful pro – and supination is the predominant complaint of patients with DRUJ dysfunction. In cases of isolated distal ulna dislocation the groove for the ECU tendon can be palpated (empty sulcus sign) [6]. Acute DRUJ instability is rather difficult to evaluate clinically due to the swelling and deformity around the wrist joint. In chronic DRUJ instability, the dorsal translation of the ulnar head is more common than the volar one, due to radial malunion [7].

## Normal MR anatomy

**Figure 2:**
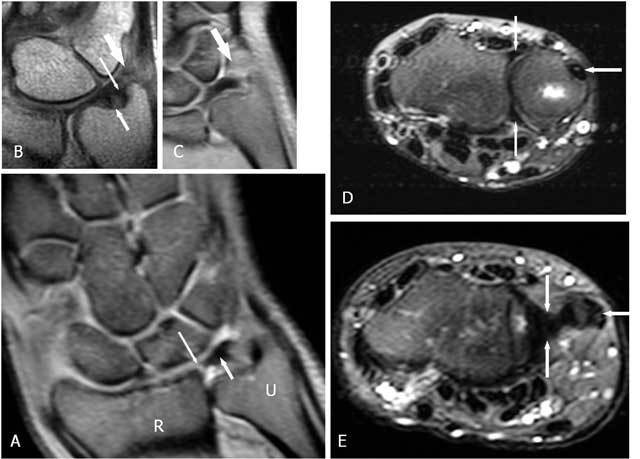
The normal components of the TFCC on coronal FS PD-WI (A), coronal T1-WI (B) and axial FS T2-WI (C and D). A. The central disc (TFC) (short arrow) is attached at the radial side to the cartilage of the radial sigmoïd notch (long arrow). B. Double insertion of the TFC at its ulnar side at the base and near the tip of the ulnar styloid (small arrow proximally and large arrow distally). The meniscus homologue (thick arrow) is and interposed between the ulnar styloid and disc proximally and the cartilage of the triquetral bone distally. C. The volar and dorsal part of the RUL (vertical arrows) are fixed between the radial notch and the base of the ulnar styloid. The m. extensor carpi ulnaris lies within his sulcus (horizontal arrow). D. The m. extensor carpi ulnaris lies on the ulnar styloid (horizontal arrow). The central disc (TFC) (vertical arrows) is attached at the radial side to the cartilage of the radial sigmoïd notch and at the ulnar side to the styloid.

**Figure 3:**
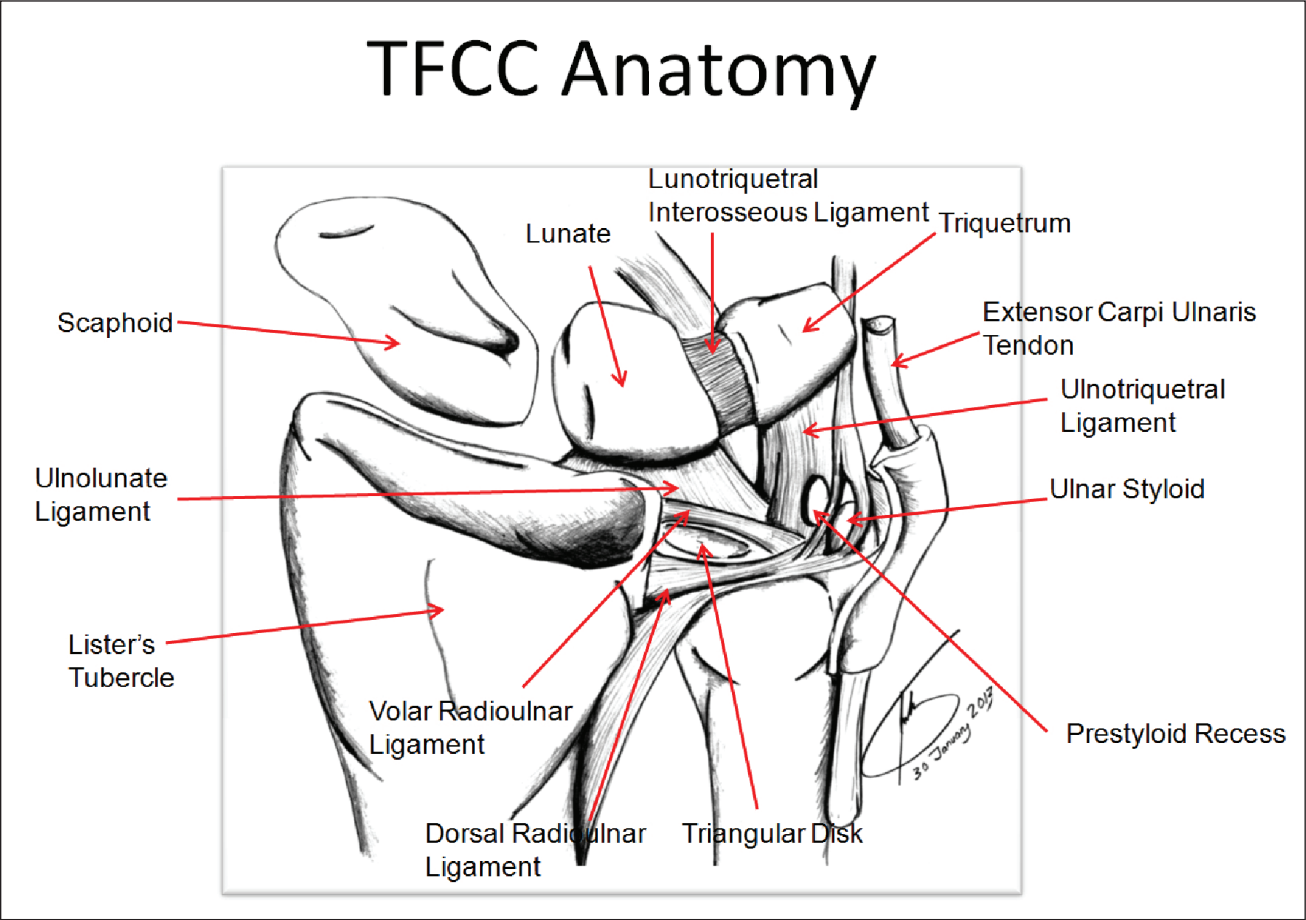
TFCC – anatomy (18): The TFCC consists of the triangular fibrocartilage, a meniscus homologue, the ulnolunate and ulnotriquetral ligament, the dorsal and palmar radioulnar ligament and the adjacent part of the sheath of the extensor carpi ulnaris tendon.

The DRUJ is a cylindrical cartilaginous joint (Fig. 1) stabilized by a complex network of soft tissue constraints, primarily components of the triangular fibrocartilage complex. The TFCC (Figs. 2, 3) [8] consists of the triangular fibrocartilage (TFC), a meniscus homologue, the ulnolunate and ulnotriquetral ligament, the dorsal and palmar radioulnar ligament and the adjacent part of the sheath of the extensor carpi ulnaris tendon. It acts as a support for the (ulnar part of the) carpus and a stabilizer of the ulnocarpal and distal radioulnar joints as bony geometry of the DRUJ contributes only approximately 20% of total joint stability [9]. It also distributes the load between the ulna and the ulnar part of the carpus, and permits forearm rotation. Furthermore the amount of articular contact at the DRUJ may vary from 60% with the forearm in neutral position to as little as 10% with the forearm at extremes of pro-supination. Therefore the joint itself has very little intrinsic stability, and relies mainly on soft tissue constraints [10].

The normal TFCC (Figs. 2, 3) consists of a central disc (TFC) with homogeneous low signal on all MR sequences (Fig. 2A and E), attached at the radial side to the cartilage of the radial sigmoid notch (Fig. 2A). At the ulnar side there is a double insertion of the TFC at the base and near the tip of the ulnar styloid (Fig. 2B). The volar and dorsal part of the radioulnar ligament (RUL) are fixed between the radial notch and the base of the ulnar styloid and firmly fixed to the anterior and posterior part of the disc. They form a hammock-like structure on axial images (Fig. 2D). The extensor carpi ulnaris tendon lies within his sulcus and on the ulnar surface of the styloid (Fig. 2D and E). The meniscus homologue (Fig. 2B and C) is attached to the ulnar collateral ligament and interposed between the ulnar styloid and disc proximally and the cartilage of the triquetral bone distally. It is of intermediate signal intensity on all pulse sequences.

The radioulnar ligament (RUL) is the strong anchor of the TFCC (Fig. 3) to the ulnar fovea (base of the styloid). The anterior RUL, in between the TFC, and the posterior RUL occur as a the already mentioned hammock-like structure. It is the major component of the TFCC and acts as a main stabilizer of this tiny joint. Both ligaments consist of two components: a superficial and a deep component (also called the ligamentum subcruentum [11]). The function of these components is different: superficial fibers of the radio-ulnar ligaments insert onto the base of the ulnar styloid, and the deep fibers insert onto the fovea. In pronation, the superficial fibers of the dorsal ligament and the deep fibers of the palmar ligament become both taut and constrain the joint. In supination the reverse occurs [9, 12]. The twisting of the RUL occurs at its foveal attachment.

## Pathology

### Radial fractures

**Figure 4:**
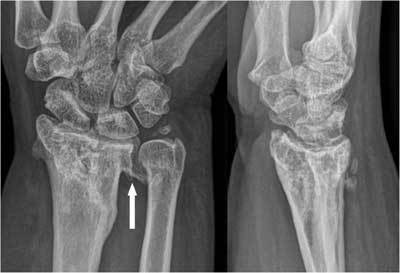
Plain radiography. Initial complex fracture of the distal radius (not shown). The follow up radiograph in this 36 y old patient shows marked premature degenerative osteoarthritis of the DRUJ after Colles’ fracture resulting in very limited and painful pro – and supination.

**Figure 5:**
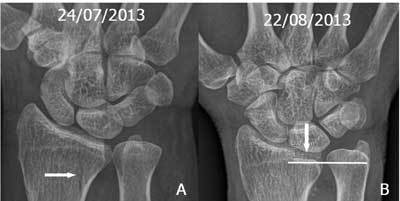
A. Initial non displaced sagittal fracture near the radial notch. B. Proximal displacement occured on the follow up radiograph resulting in deformity of the DRUJ and subluxation.

Distal radial fractures that may cause DRUJ dysfunction include Colles’ fracture, Smith ‘s fracture, Barton ‘s fracture and Chauffeur ‘s fracture. The cause of the most common Colles’ fracture is a fall on an outstretched hand (FOOSH) [13]. The upper limit of an acceptable deformity after reduction of the fracture is 20° of dorsal tilt. When dorsal tilt beyond the acceptable threshold occurs, DRUJ motion is also altered, and forearm rotation becomes restricted. Distal radius fracture lines often extend into the DRUJ and are present in 55% of dorsally angulated intra-articular fractures [14]. In younger patients, intra-articular fractures of the distal part of the radius are often the result of high energy trauma and may be associated with disruption of the DRUJ. Instability of this DRUJ is an important cause of ulnar-sided wrist pain in distal radius fractures [15]. They are also associated with a high frequency of post-traumatic degenerative osteoarthritis. In fractures healing with residual discongruity in a study although of the radiocarpal joint [16] degenerative osteoarthritis (Fig. 4) occured in 91 %, whereas fractures that healed with a congruent joint, degenerative osteoarthritis developed in only 11 %. The shape and/or position of the radial notch may evolve during the healing process in fractures near or through the DRUJ (Fig. 5). Even minor displacement and/or deformity may cause joint dysfunction. Clinical symptoms such as persistent pain, motion restriction and instability at the DRUJ call for further investigation in case of clinical suspicion of DRUJ dysfunction. Therefore meticulous follow up by plain radiographs is recommanded.

**Figure 6:**
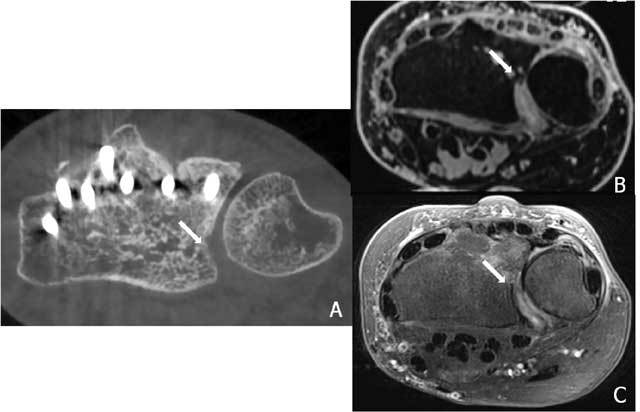
Additional value of cross sectional imaging in the evaluation of the DRUJ congruity A. Axial CBCT: the evaluation is not disturbed by the metal on CBCT. Small step off at the DRUJ. B. Axial 3D – GRE and C. Axial FS T2-weighted imaging Axial : discongruity and thinning of the cartilage is directly visible on MRI.

Due to its complex anatomy, analysis of the DRUJ is incomplete on radiography. Particulary coronal plane fractures entering the sigmoid notch may be difficult to identify. Bony discongruity (Fig. 6) can better be evaluated by CT-scan (CT) or by the recently introduced cone beam (CBCT) for extremities [17], than by magnetic resonance imaging (MRI). CBCT is even much less disturbed by metallic parts [18]. Another advantage of CBCT is the significant lower dose of radiation than conventional CT and reported to be 6 to 10 times lower than MDCT [19]. The isotropic spatial resolution is up to 0,1 mm. Sub-millimetric isotropic voxels can recently also be obtained with 3.0-T MRI [20].

**Figure 7:**
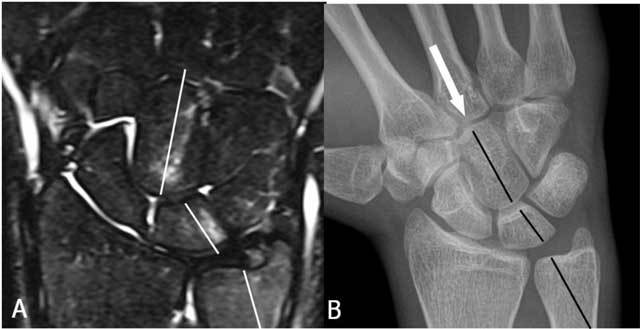
Unraveling the trauma mechanism by analysis of the BMO pattern: A. Coronal FS T2-WI: the BMO pattern on the T2-FS WI (white lines ) runs through the capitate bone, the lunate bone and the distal ulna. B. Plain radiography: alignment (black lines) suggests a distal impaction trauma in maximal radial deviation.

For the evaluation of the cartilage MRI / MR-arthrography (MR(A)) or CT-arthrography (CTA) is needed for detailed evaluation of the thickness and the surface of the cartilage. Occult bone trauma and unravelling the mechanism of trauma: MRI is a very useful technique to document bone marrow oedema [21, 22]. Meticulous analysis of the bone marrow oedema (BMO) pattern may reveal the mechanism of trauma [23]. This information may be important in surveyor’s reporting to determine whether the trauma is related to work or not. The correlation between the mechanism of trauma and the detected lesion(s) often is unclear on clinical examination and on plain films. MRI can illustrate an oedema pattern [24] (Fig. 7) and possibly clarify the relation between the trauma mentioned by the patient and the apparent lesion(s).

## (Sub)luxation of the DRUJ

**Figure 8:**
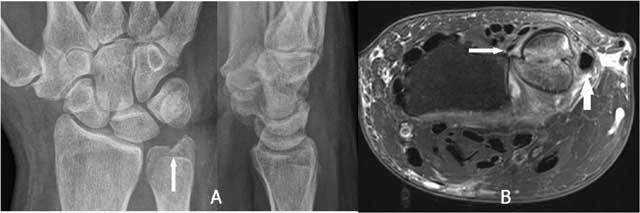
A. Plain radiography: the double contour image of the ulnar head may raise suspicion of DRUJ subluxation. B. Axial FS T2-WI shows a dorsal subluxation of the ulna with a coronal fracture (small arrow) and palmar luxation of the ECU (thick arrow).

(Sub)luxation of the ulna at the DRUJ may occur in dorsal and less frequently in palmar direction. Dorsal (sub)luxations (Fig. 8) result from fall on pronated hand. Clinically, prominence of ulnar head and loss of supination is seen. An injury to the TFCC is part of this injury.

Some are associated with entrapment of the tendon of the m. extensor carpi ulnaris (ECU), which will cause problems for the closed reduction. This dorsal luxation may occur as an isolated injury or in association with other (more complex) fractures (Colles, Essex-Lopresti, Galeazzi fracture). The volar subluxation occurs less often and results from forced supination.

**Figure 9:**
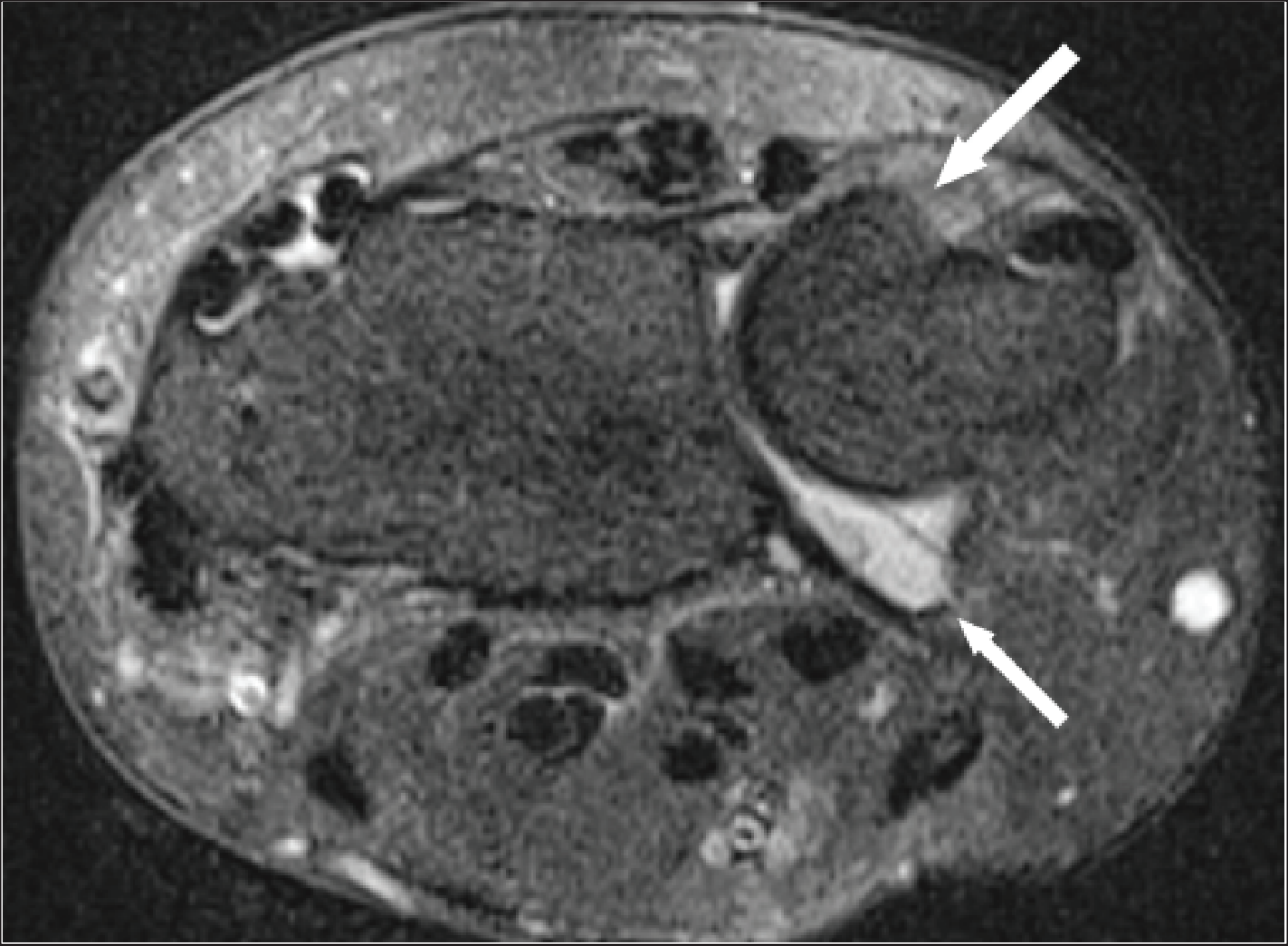
Posttraumatic contour deformity after DRUJ dislocation. FS T2-WI Axial. A bony contour deformity (large arrow) persists after reduction of an anterior dislocation of the ulna. The anterior part of the capsule is distended and filled with joint fluid (small arrow).

**Figure 10:**
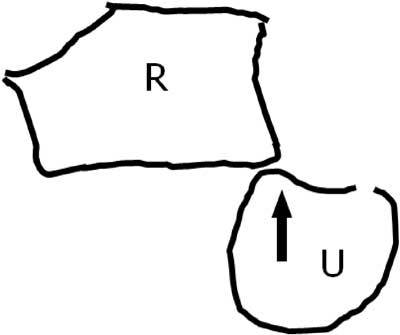
Mechanism of the trauma. Palmar luxation of the ulna (U) with impaction of the anterior side of the radial notch in the ulna. R = radius.

Standard radiograph may be straightforward in case of overt dislocation, but (CB)CT in pronation and supination may be necessary to evaluate more subtle dislocations or subluxations. The entrapment of the ECU can be suspected clinically and confirmed by dynamic ultrasound. In few cases a posttraumatic contour deformity similar to a ‘Hill-Sachs lesion” (after anterior shoulder dislocation) can be found at the distal ulna after reduction in anterior luxation (Figs. 9, 10).

### Ulnar styloid fractures

Fractures of the ulnar styloid process are often associated with fractures of the distal radius and TFCC tears. No significant instability of distal radioulnar joint appears when the distal part of the ulnar styloid process is involved as the TFCC inserts at a small distance from the tip distally and at the base to the ulnar notch (Fig. 2B). On the contrary ulnar styloid fractures at the base disrupt the major stabilizing ligaments of the distal ulna and TFCC and may lead to instability of the DRUJ [25, 26, 27].

**Figure 11:**
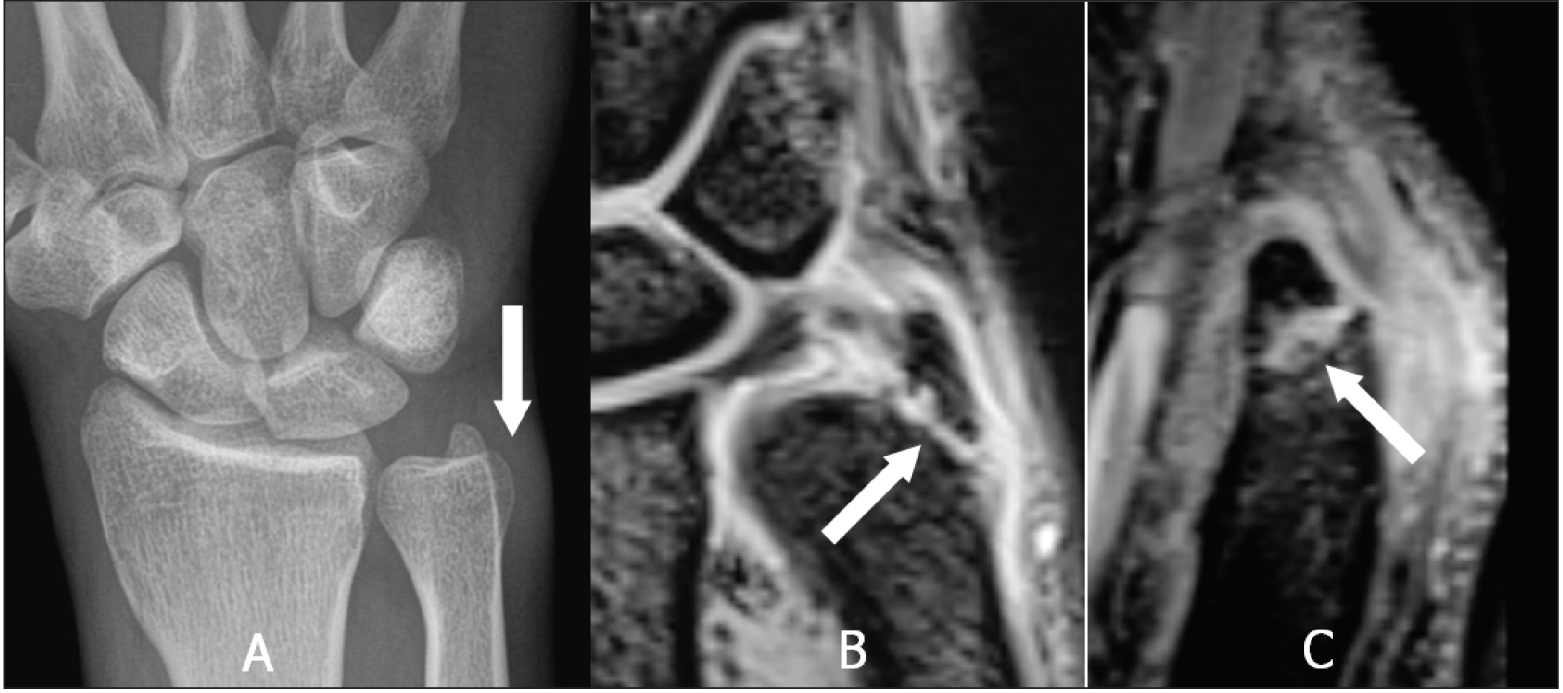
Non displaced fracture at the base of the styloid process of the ulna. A. Plain radiography: although there is a suspicion of a fracture as the peristyloidal fat line is blurred and there is adjacent soft tissue swelling, direct visualisation of the fracture line is not possible due to the double oblique course. B. and C. 3D-GRE Coronal and Sagittal: demonstrates clearly an oblique fracture at the base of the styloid.

**Figure 12:**
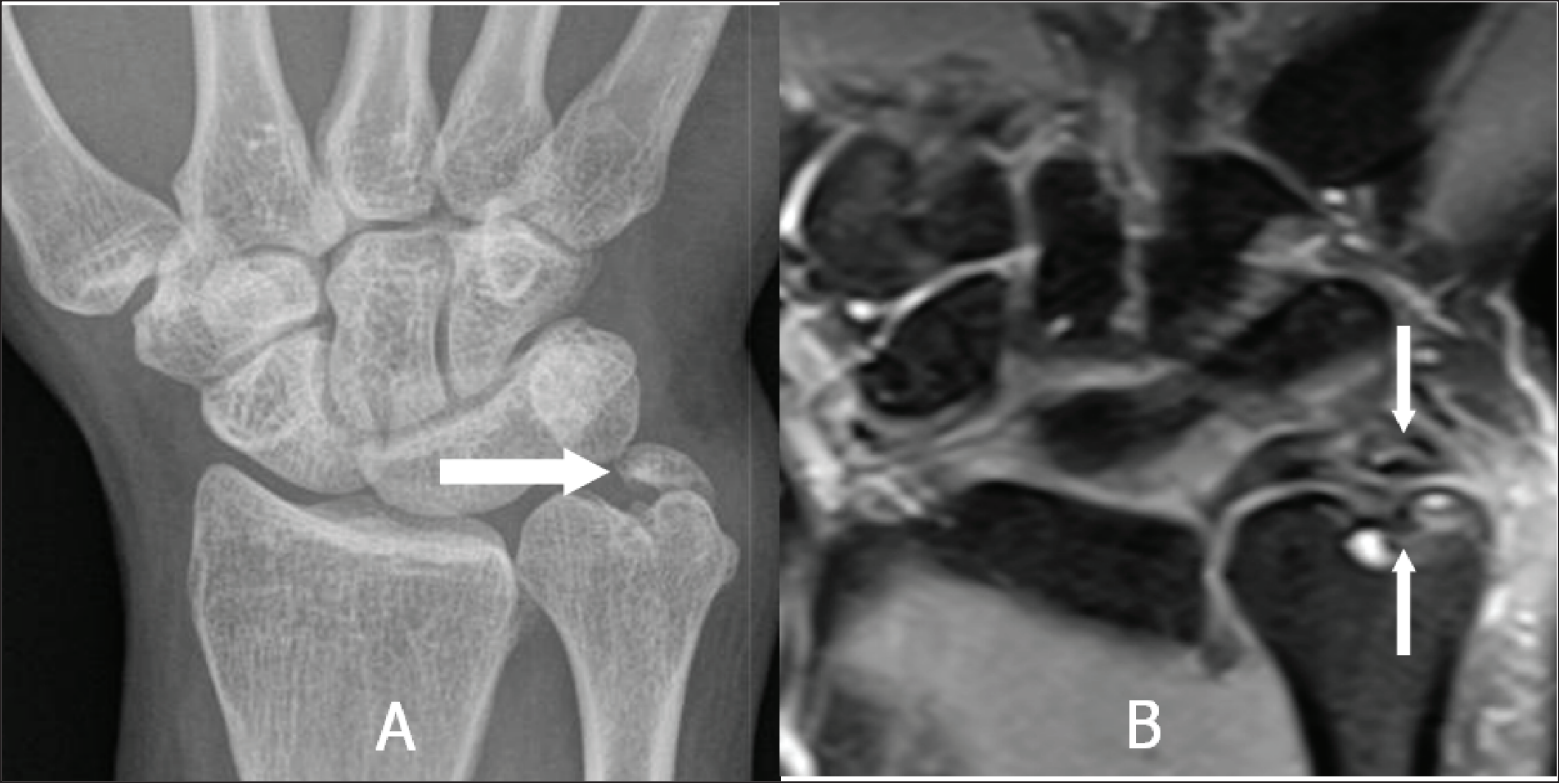
Pseudarthrosis of the ulnar styloid process. A. Plain radiography: neo-articulation near the base of the styloid (white arrow). B. FS T2-WI: bone marrow oedema and degenerative subcortical cysts.

**Figure 13:**
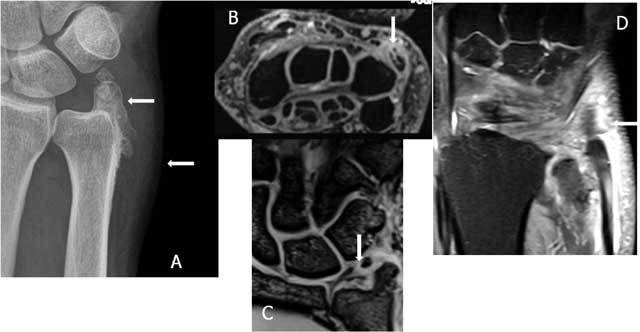
Posttraumatic deformity of the ulnar styloid. A. Plain radiography: obvious deformation of the ulnar styloid with a sharp bony ridge. The surrounding soft tissue is swollen (white arrows). B. Axial MR 3D GRE shows a fissuration of the ECU, on (C) 3D GRE Coronal) a I-B lesion of the TFCC C and on (D) Coronal FS T2 WI a tenosynovitis of the ECU.

**Figure 14:**
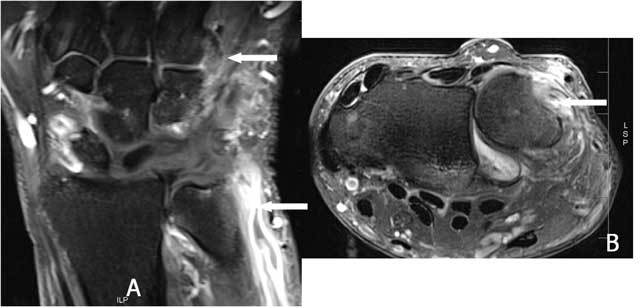
Complete rupture of the ECU. Coronal (A) and Axial (B) FS T2 WI: complete rupture with retraction of the ECU tendon and an empty sulcus sign.

**Figure 15:**
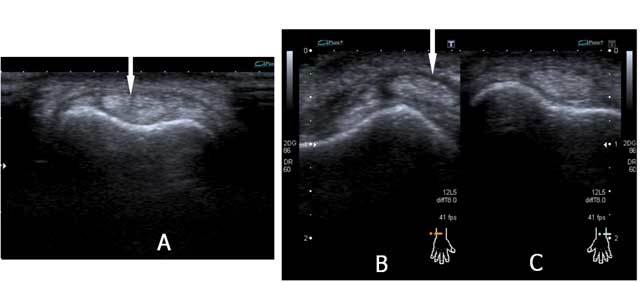
Fissuration and (dynamic) luxation of the ECU: Ultrasound. A. Fissuration (white arrow) of the ECU. B. The wrist is examined in supination (B) and pronation (C) to evaluate dislocation that is only visible on dynamic examination. The tendon dislocates from the sulcus in wrist supination (B) and is in its normal position in pronation (C).

**Figure 16:**
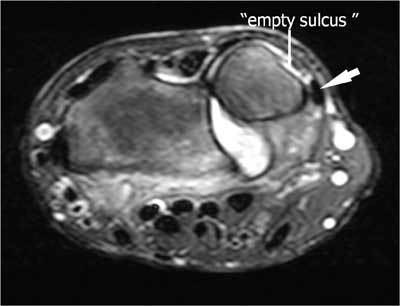
Empty sulcus sign (small arrow) due to palmar luxation of the ECU (large arrow) on an axial FS T2-WI.

**Figure 17:**
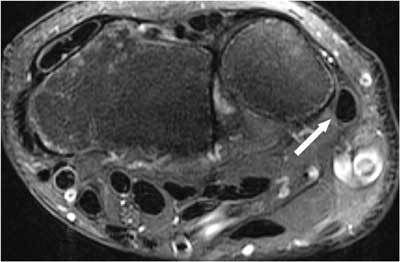
Tendon dislocation (ECU) and rupture of the extensor retinaculum (white arrow) on axial FS T2-WI.

On plain radiographs specific attention should be given to the peristyloidal fat line (Fig. 11) in order to detect subtle (hair line) fractures and eventually additional views should be added. As these fractures, especially when localised near the base or orientated in a (double) oblique direction, can be easily missed on conventional radiography, (CB) CT and even MRI (Fig. 11) should be considered if there is a high index of clinical suspicion. Focal persistent pain at the ulnar styloid after “fracture healing” should prompt for MRI to exclude symptomatic pseudarthrosis (Fig. 12). Recent fractures have irregular or ragged margins [2]. The borders of ossicles however are smooth and corticated. An old styloid fracture should be differentiated from a lunula, which is an accessory ossicle that lies in the TFCC, between the styloid process and the triquetral bone. It may be fused with the styloid process, giving it an elongated appearance. An os triangulare is situated more radial of the styloid process. Posttraumatic deformity of the ulnar styloid (Fig. 13) is a predisposing factor for dislocation (Fig. 7B), partial tearing (Fig. 13) and even complete rupture (Fig. 14) of the ECU. US should be the first choice of examination. The ECU mostly dislocates toward the palmar side (Fig. 7B), but dorsal dislocation is possible. Examination should be performed in pronation and supination to exclude a dynamic dislocation (Fig. 15). The “empty sulcus sign” (Fig. 16) is characteristic in case of dislocation or rupture of the ECU. The wrist should always be mobilised to evaluate the position of the ECU (Fig. 15B, C). Integrity or rupture (Fig. 17) of the extensor retinaculum may be evaluated as well.

### Positive ulnar variance – ulnar impaction syndrome

Distal radius fractures typically result in loss of length as the radius due to extensive comminution and impaction of fragments into the metaphysis. This is more disabling than an angular deformity of the distal radius and may lead to an acquired positive ulnar variance, ulnar impaction syndrome and DRUJ instability. Ulnar impaction syndrome is a painful condition of excessive contact and wear between the ulna and the carpus and may be associated with a degenerative tear of the TFCC Often the patient will present with significant loss of pronation and supination. The ulnar length is evaluated on a strict postero-anterior radiograph using the Hulten criteria [28]. The ulnar variance refers to the relative lengths of the distal articular surfaces of the radius and ulna. Therefore a line is drawn tangent to the radial side of the DRUJ, perpendicular on the radial axis. Neutral variance means that they are both of the same length, positive that the ulna is longer and negative that the ulna is shorter.

In the pediatric population the distal radius is the most frequent injured site of all physeal injuries (28%) [29]. Sometimes the relative length of the ulna can evolve from a negative to a positive ulnar variance (Fig. 18) in cases of radial epiphysiolysis or Salter-Harris fracture [30]. Approximately 15 % of all pediatric fractures involve the physis. Nearly 15 % of all physeal fractures lead to growth arrest with formation of bone bridge across the physis causing shortening or angular deformity [31]. Significant positive ulnar variance will contribute to the limitation of the radiocarpal mobility of the wrist (Fig. 19). Bony alterations (subchondral cyst(s), sclerosis can be observed at the proximal-ulnar side of the lunate bone only in a late fase of ulnar impaction. Early diagnosis will be revealed by MRI.

**Figure 18A:**
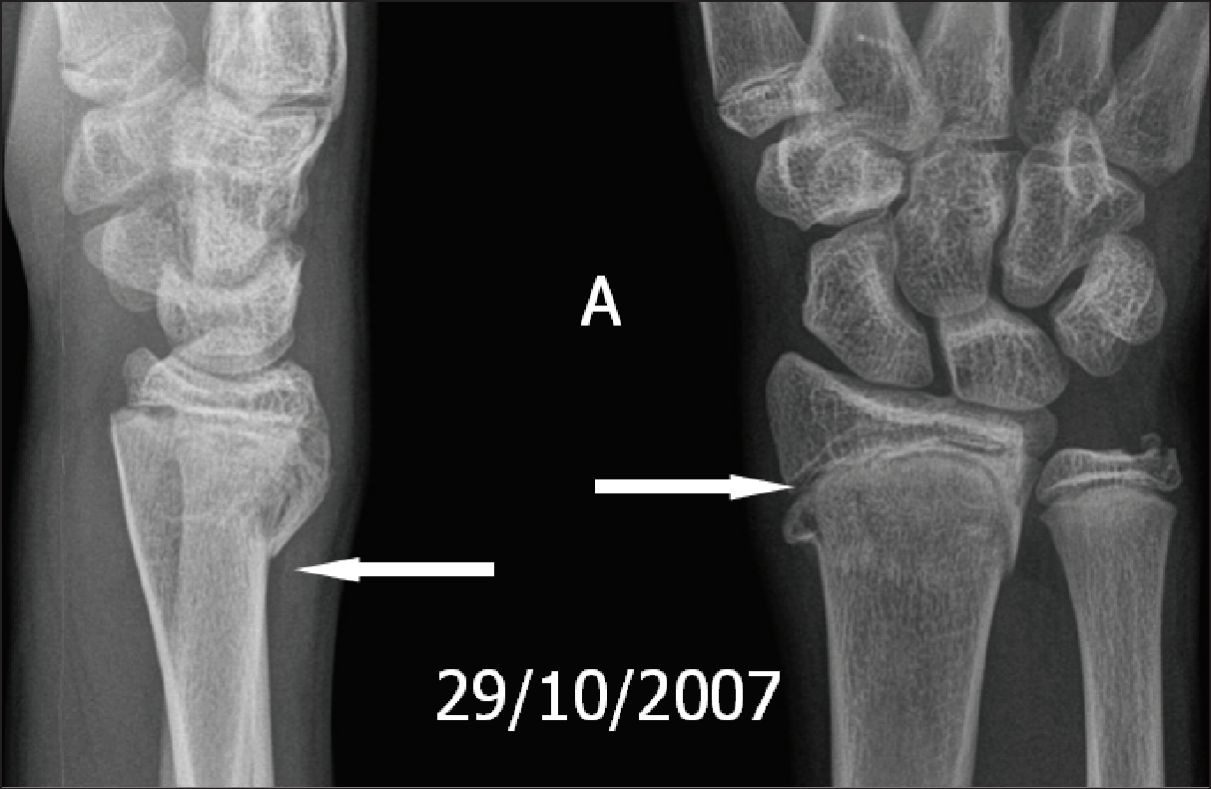
Standard radiograph. Salter-Harris fracture type 2 at the distal radius.

**Figure 18B:**
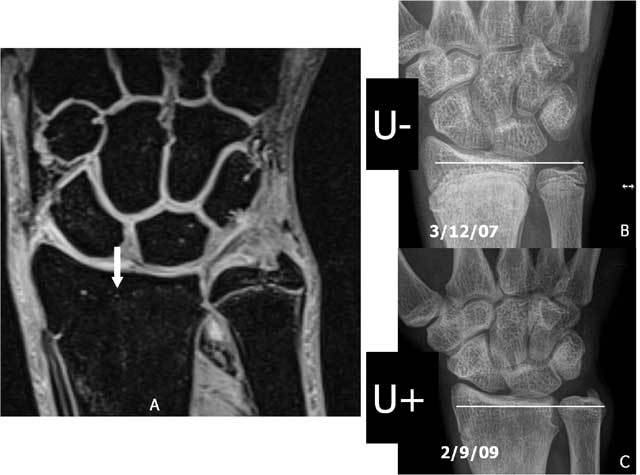
Premature closure of the physis of the radius due to Salter-Harris fracture. A. 3D-GRE Coronal shows a premature closure of the distal physis of the radius. B. Plain radiography: negative ulnar variance (U−) at the moment of the initial healing process. C. Plain radiography: the ulnar length “increased” to a positive ulnar variance (U+) due to the premature closure of the radial physis.

**Figure 19:**
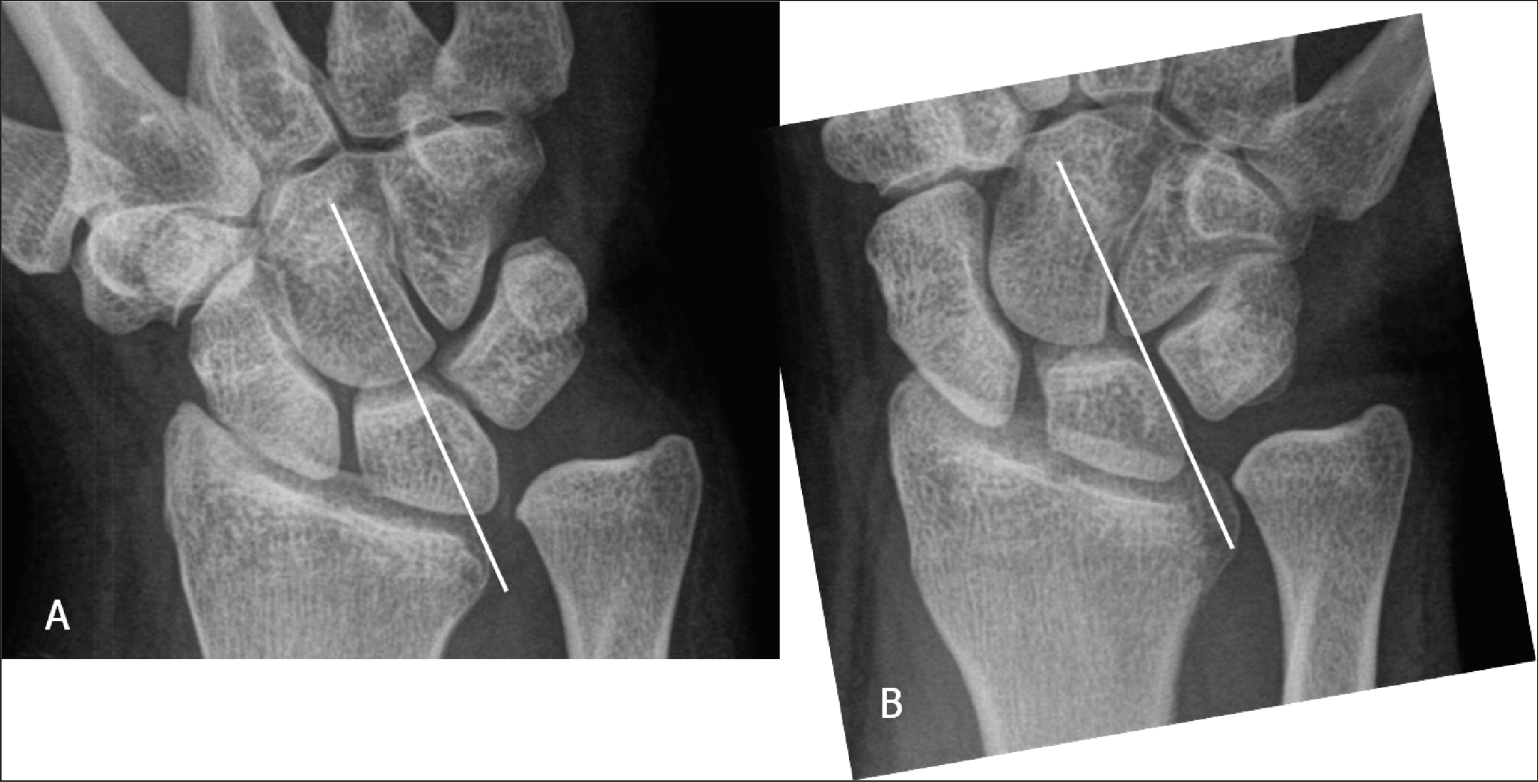
Marked positive ulnar variance and DRUJ luxation. Plain radiography in radial (A) and ulnar deviation (B): the lunate bone shows only a very moderate motion in relation to a tangent line to the deformed radial notch due to limited radiocarpal motion.

### TFCC-tears and (sub)luxation of the TFC

**Figure 20:**
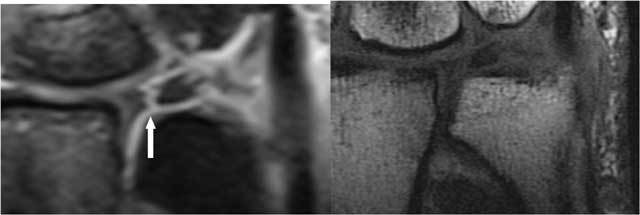
Acute TFCC tear on FS T2-WI (A) and T1-WI (B).

Acute TFCC injuries (Fig. 20) may occur after a fall on an outstretched hand (FOOSH) with the wrist in extended and pronated position, or from a forceful rotation or distraction. They are commonly found in conjunction with distal radius fractures, being reported in up to 35% of intra-articular fractures and in 53% of extra-articular fractures [32]. Especially in patients with complete peripheral TFCC tears DRUJ instability will appear in a very large number (+/– 90 %) [33] at the follow-up examination.

**Figure 21:**
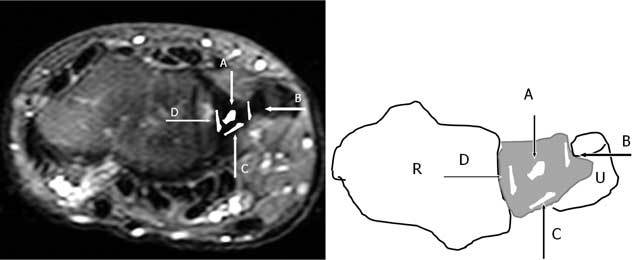
Axial FS T2-WI. The Palmer classification for traumatic lesions (type I): A: central perforation, B: ulnar avulsion with or without distal ulnar fracture, C: distal avulsion from the carpus, D: radial avulsion with or without sigmoid notch fracture. R: Radius, U: Ulna.

**Figure 22:**
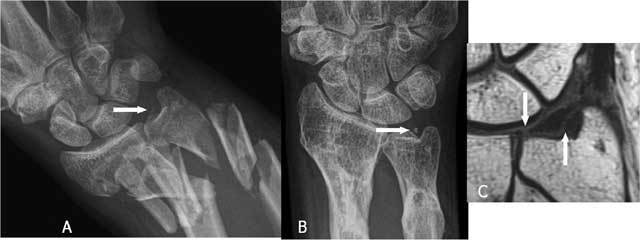
A very small loose, cortical delineated fragment near the ulnar styloid may suggest a TFCC lesion in major trauma. A. Plain radiography: complex fracture of ulna and radius with a barely visible fragment (arrow) near the ulnar styloid. B. Plain radiography after fracture healing: a small, cortical delineated fragment near the ulnar styloid (arrow) becomes more obvious. C. Coronal T1-WI: TFCC tear I A and I B (arrows).

**Figure 23:**
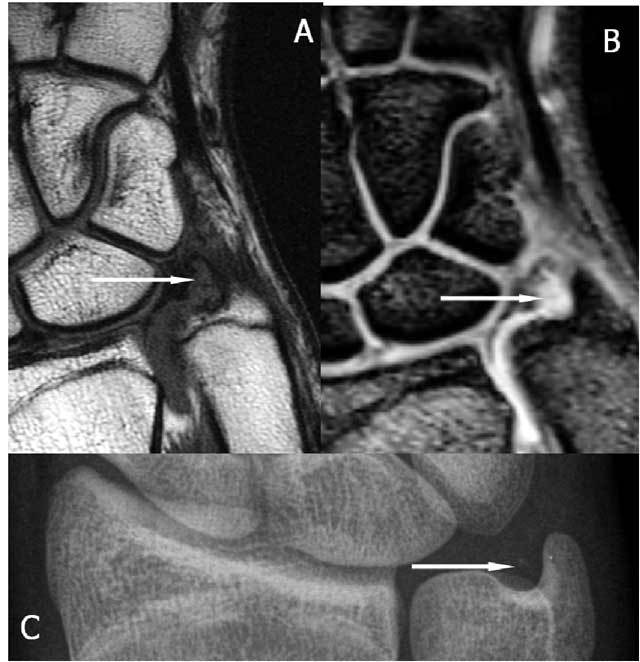
A very small loose, cortical delineated fragment near the ulnar styloid may suggest a TFCC lesion in trauma without other major radiological lesions. A. Plain radiography: a small, cortical delineated fragment near the ulnar styloid (arrow) is present. B-C. Notice a TFCC 1B tear (arrow) on coronal T1-WI (B) and coronal FS T2-WI (C).

The Palmer classification (Fig. 21) for traumatic lesions [34], the so called Palmer I types (A: central perforation, B: ulnar avulsion with or without distal ulnar fracture, C: distal avulsion of the volar ulnocarpal ligaments (ulnolunate and/or ulnotriquetral ligament) and D: radial avulsion with or without sigmoid notch fracture), mainly consists of A and B types. A very small loose, cortical delineated fragment can indirectly suggest a TFCC lesion. In case of complex trauma, they are often overlooked (Fig. 22). In isolated appearance (Fig. 23) particular caution should be made for small calcifications which appear more frequently in the TFCC region and are difficult to differentiate from avulsion fractures. MRI can be used for initial evaluation and is sufficient in many cases. It has proven to be reliable in central and radial-sided TFCC tears, but showed worse results for peripheral attachment tears [35].

**Figure 24:**
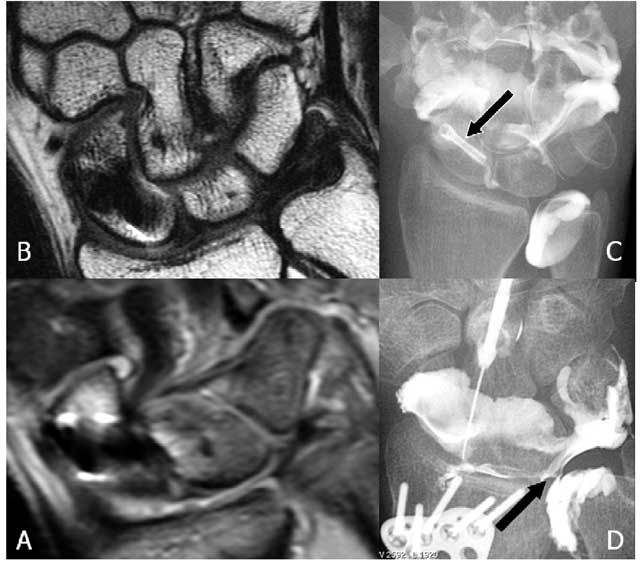
Arthrography remains a useful examination for specific TFCC lesions. A: Coronal FS T2-WI and B: Coronal T1-WI : artifacts due to the small Herbert screw (black arrow) are mainly in the region of the scapholunate ligament. C. Mediocarpal and distal radio-ulnar arthrography show a normal TFCC. No interaction of the old Herbert screw in the scaphoid bone. D. Radiocarpal arthrography (other patient): larger osteosynthesis plate is present. Metallic artifacts do not interfere with arthrography. A central TFCC I A lesion is seen (black arrow).

**Figure 25:**
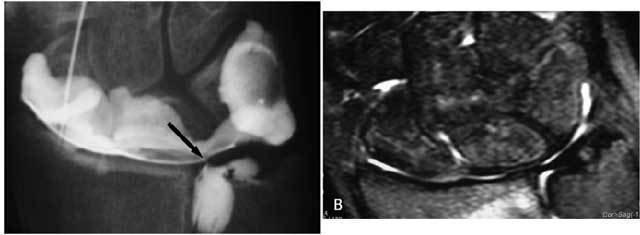
A. Radiocarpal arthrography shows a central hairline fissure of the TFCC. B. MRI is unable to detect the lesion.

**Figure 26:**
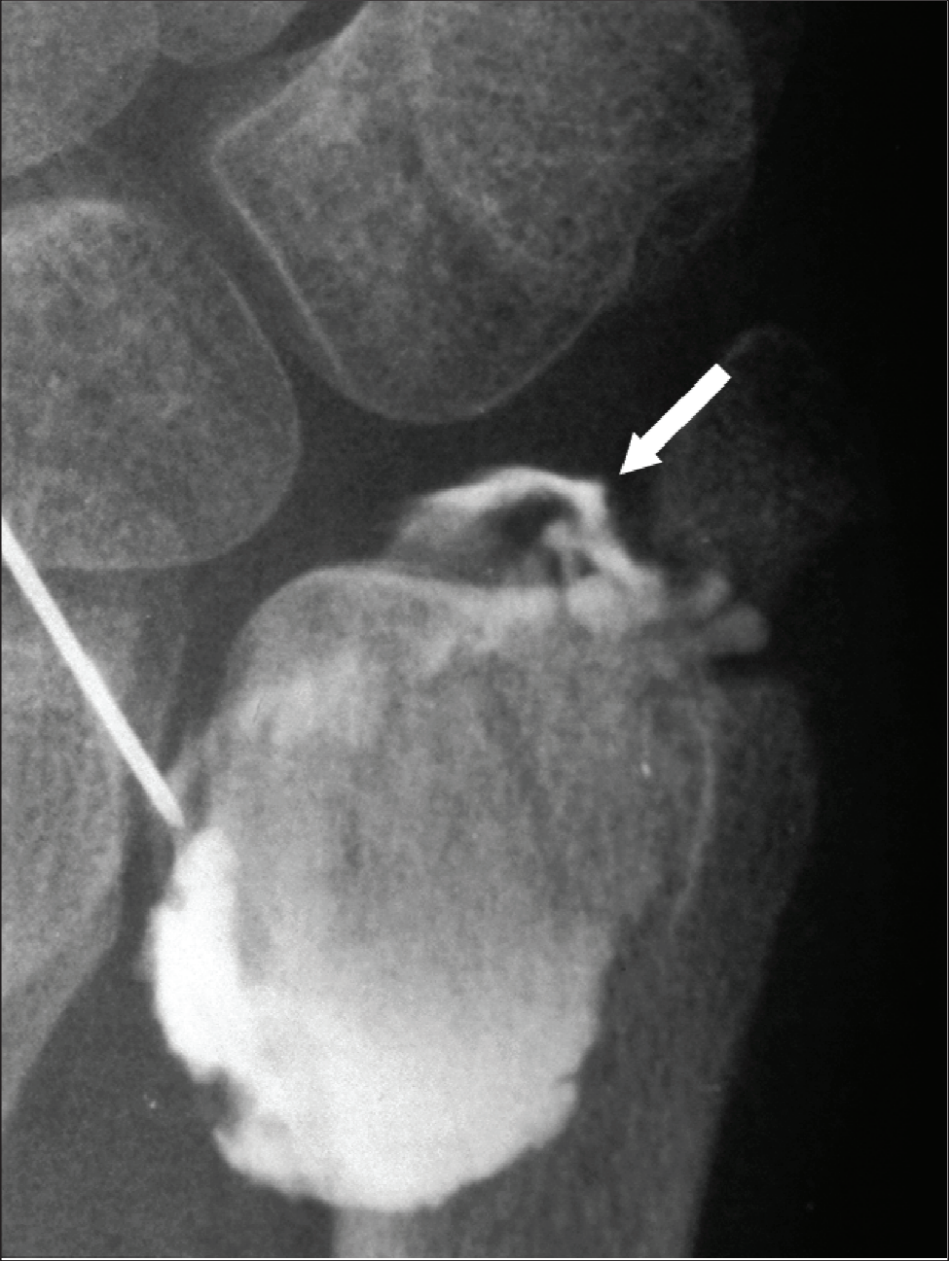
Distal radio-ulnar arthrography reveals a Palmer type I-B lesion.

However direct MR arthrography (MRA) is – in comparision with standard MRI – more sensitive in the specific detection of surface lesions of the cartilage [36] and is substantially beter for the evaluation of the TFCC [27]. Lack of spatial resolution can be a limiting factor especially in concave or convex structures. Multi detector CT arthrography (MD-CTA) is a much faster technique and has excellent spatial resolution. Moreover multiplanar reformats make it even more useful. It offers an equal sensitivity and specificity for the TFCC lesions than MRA [37, 38]. MRA or MD-CTA is sufficient for the evaluation of most of all the soft tissue lesion(s). With the advent of cross-sectional imaging techniques, conventional arthrography is most frequently undertaken as a part of MRI or CT arthrography. However conventional arthrography images remain valuable in exceptional cases [39]: presence of a metallic osteosynthesis at the wrist (Fig. 24) (less artefacts than MD-CT), to detect central hair line I A tears of the TFC (Fig. 25) (still higher resolution than MD-CT) and a distal radio-ulnar arthrogram in cases of on MRA/MD-CTA or clinically suspected very destabilising I B lesions (Fig. 26) (evaluation of dynamic changes during stress views).

**Figure 27:**
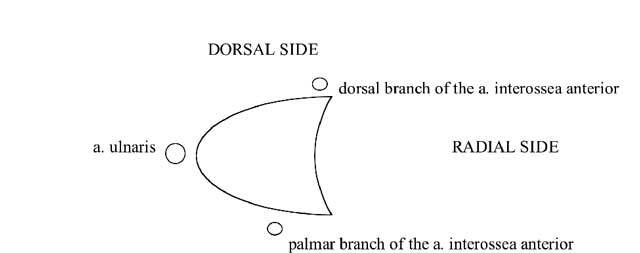
The vascular supply of the TFCC by three arterial branches (a. ulnaris and a. interossea anterior with a palmar and dorsal branch). In the peripheral vascular zone (white zone) of the TFFC tears can heal, in contrast with tears in the central avascular zone (grey zone).

**Figure 28:**
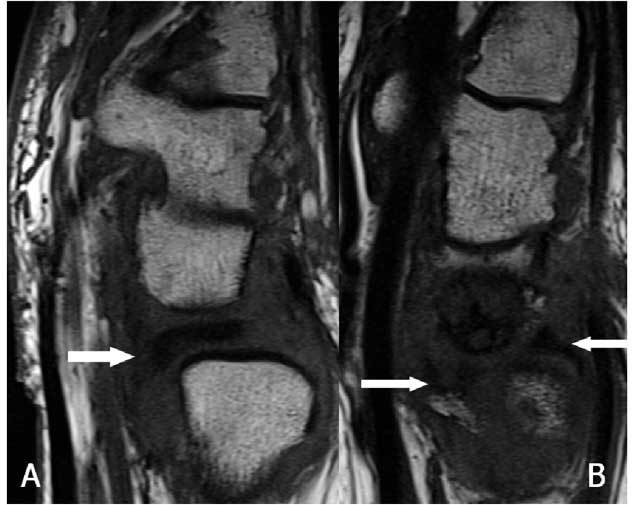
Anterior subluxation and fragmentation of the TFC. A. Sagittal T1-WI: the disc is dislocated towards the palmar side (arrow). B. Sagittal T1-WI: the disc is split due to an impaction with migration of the fragments in anterior and posterior direction (arrows).

**Figure 29:**
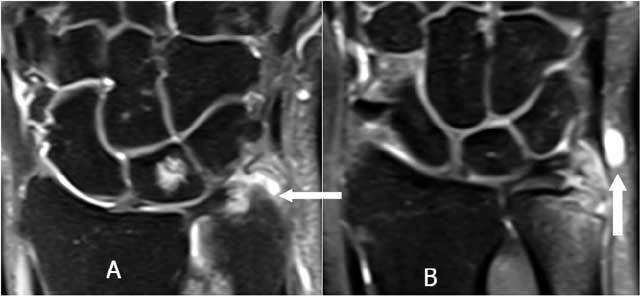
Coronal FS T2-WI (A): a normal prestyloidal recessus and synovial cyst formation due to a leak of the capsule (B).

**Figure 30:**
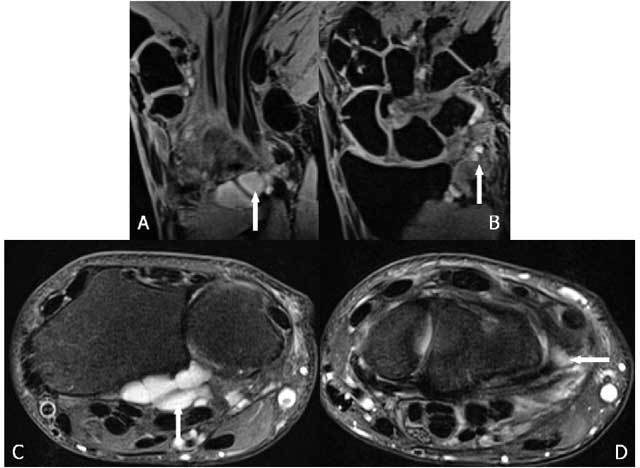
Adjacent cyst formation (arrow) in a TFCC I B tear on coronal 3D-GRE (A, B) and axial FS T2-WI (C, D).

It is very important to evaluate whether the avascular central zone of the TFCC (Figs. 20, 23, 24) or the vascular peripheral ulnar zone (Figs. 22, 25) is involved. The TFCC is supplied by three arterial branches (a. ulnaris and a. interossea anterior with a palmar and dorsal branch). In the peripheral vascular zone (Fig. 27, white region) of the TFFC tears can heal due to the denser vascularity, in contrast with tears in the central so called avascular zone (Fig. 27, grey region) where no spontaneous healing can occur. Additional (sub)luxation and fragmentation of the TFC (Fig. 28), capsular leakage with cyst formation (Fig. 29) or adjacent TFCC cysts (Fig. 30) may be evaluated as well.

### Specific soft tissue lesions of the DRUJ

**Figure 31:**
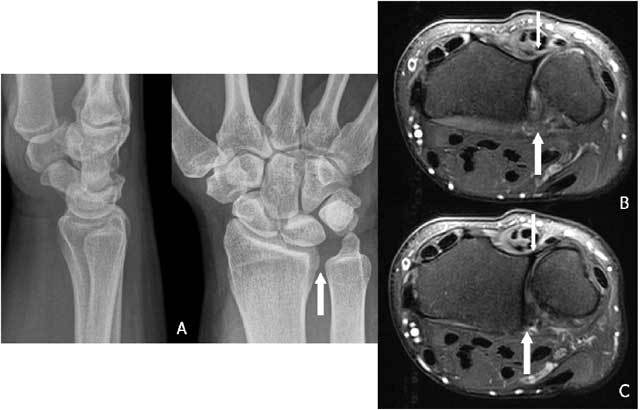
DRUJ instability with RUL rupture. A. Plain radiography: diastasis at the DRUJ (arrow). B–C. Axial FS T2-WI: tear of the palmar RUL (large arrow) with an intact dorsal RUL (small arrow).

– a. anterior / posterior DRU – ligament (part of TFCC, see TFCC tears) When the RUL is torn (Fig. 31), instability of the DRUJ occurs [40]. Demonstration of RUL laceration remains a radiological challenge even for MR(A) or CTA.

– b. capsule retraction: The capsule is a secondary soft tissue stabilizer of the DRUJ, as well as the interosseous membrane, the extensor carpi ulnaris and its deep part of the sheath and the m. pronator quadratus. The capsule of the DRUJ allows pronation and supination and also limits the maximum range of motion [41]. The dorsal part of the joint capsule is extended in pronation and folded in supination. On the contrary, the palmar part of the joint capsule is folded in pronation and extended in supination. The normally large capsule allows this motion and a retraction due to posttraumatic fibrosis will therefore limit significantly any motion of the DRUJ [41, 42].

**Figure 32:**
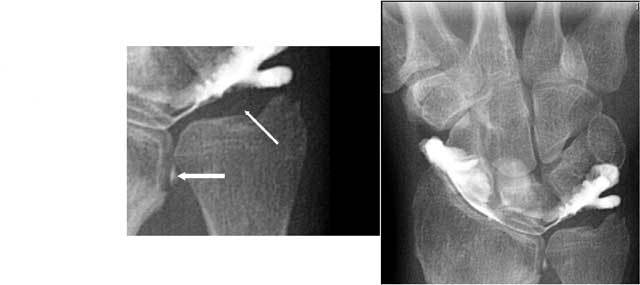
Radiocarpal arthrography shows a retraction of the capsule of the DRUJ with only very faint opacification (arrows) through a TFCC I A tear. There is also a rather small capacity of less than 1,5 cc of the radiocarpal joint (normally 2–3 cc).

**Figure 33:**
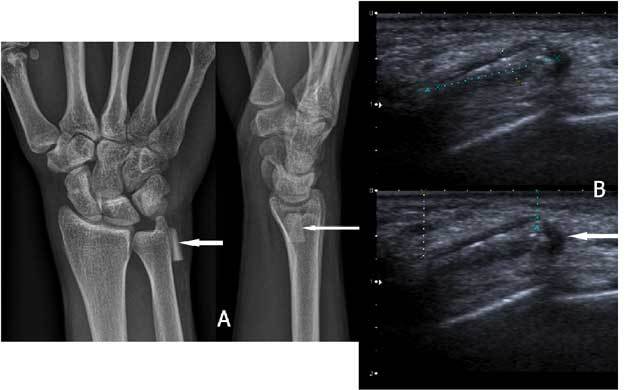
Painful swelling after old trauma and previous removal of glass fragments: foreign body (glass fragment) still present. A. Plain radiography: opaque structure adjacent to the distal ulna (arrow). B. Ultrasound. Longitudinal image: linear hyperechogenic structure surrounded by a hypoechogenic halo in the subcutaneous fat (arrow).

This pathology remains one of the residual indications for conventional arthrography of the DRUJ (Fig. 32). Forceful injection and extensive mobilisation after the contrast injection into the radiocarpal joint is mandatory to differentiate from insufficient filling. Direct injection into the DRUJ (normal content of 0,5–1,5 cc) is needed if the TFCC is intact but is often difficult due to the capsule retraction. 8/ Peri-ulnar foreign bodies and soft tissue laceration: In a defensive reaction against trauma or violence the forearm and wrist or often forwarded as a first reflex motion. Some foreign bodies, such as small glass fragments, sometimes become embedded and can cause painful motion when they are in the vicinity of the ECU or the DRUJ capsule. In most cases X-ray and ultrasound (US) are able to evaluate the presence and extent of the foreign body (Fig. 33) [43]. Hematoma and abscess formation are often concomitant findings.

**Figure 34:**
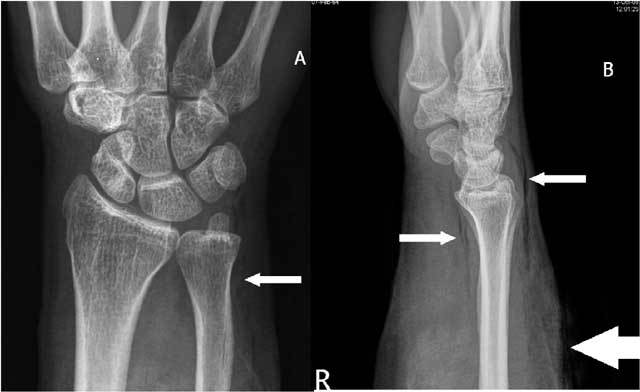
Anaerobic infection due to animal bite. Plain radiography A. PA B. Lateral view: the treated wound of the distal forearm (thick arrow) from repeated bites of a dog started to swell and showed crepitations due to gas formation around the wrist at a distance from the wound (smaller arrows) deep under the subcutaneous fat along the m. pronator. quadratus (palmar side) and the extensor tendons (dorsal side).

Dog bites [44] around the ulna are a risk factor for development of infection around and arthritis of the DRUJ. Laceration of the ECU tendon is best evaluated by US. Caution should be given to gas formation in the small parts at a distance from the wound as they may be suspicious for anaerobic infections (Fig. 34) [44, 45].

## Conclusion

A large variety of pathologic conditions may involve the DRUJ. This underscores the need for a thorough evaluation of the small distal radio-ulnar joint as almost every fine three dimensional motion of the hand – wrist combination includes a pronation and/or supination component at this joint.

Also concerns about the long term prognosis – certainly in expert or insurance related files – should encourage detailed assesment of this joint, including the evaluation of the soft tissues as even small lesions can be very arthrogenic and functionally disabling on the long term.

## Competing Interests

The authors declare that they have no competing interests.
